# P232: Prospective surveillance of intracranial infection in neurosurgical intensive care unit

**DOI:** 10.1186/2047-2994-2-S1-P232

**Published:** 2013-06-20

**Authors:** N Kurdumova, G Danilov, O Ershova, M Shifrin, T Tabasaranskiy, I Savin, E Sokolova

**Affiliations:** 1 ICU, Burdenko Neurosurgery Institute, Moscow, Russian Federation; 2 Neurosurgery, Neurotraumatology, Burdenko Neurosurgery Institute, Moscow, Russian Federation; 3 Epidemiology, Burdenko Neurosurgery Institute, Moscow, Russian Federation; 4 Medical IT Laboratory, Burdenko Neurosurgery Institute, Moscow, Russian Federation

## Introduction

Intracranial infection (ICI) is a dangerous complication for neurosurgical patients at the intensive care unit (ICU). A comprehensive information about the infection rates and risk factors is not available.

## Objectives

The prospective study was conducted to establish the ICI rates and risk factors for patients at Neuro-ICU.

## Methods

All the data on patients staying in Neuro-ICU for more than 48 hours in 2010 - 2012 (30 months) was daily entered into a database. These data included the use of mechanical ventilation, intravascular devices, external ventricular/lumbar drains, urinary catheters, Levin tubes etc. The presence of intracranial, respiratory, blood and urinary infections was considered based on the CDC Definitions of Nosocomial Infections (2008). All the data were analyzed statistically.

## Results

The collected data for a total of 777 ICU patients with neurosurgical pathology were saved in the electronic database.

Patients with ICI (109 pers.) showed the same distribution of the main neurosurgical pathology (p = 0.7) and gender structure (p = 0.08) as the group of other cases. The differences were found in patients’ age (average 35.2 years and 42.1 years respectively, p = 0.0003).

The ICI rate was 14% ± 1,2% (CI 11.65% - 16.35%). In this group of patients bloodstream infections occurred significantly more often (p = 0.002), as well as respiratory (p = 0.035) and urinary tract infections (p <0.000) were more common. Significant differences were observed in the use of external ventricular drainage (p <0.000). No differences were seen in the use of intracranial pressure monitoring (26.6% in the studied group versus 20.1%). The presence of ICI significantly prolonged the length of stay in ICU (an average of 38.9 days versus 18.1 days, p <0.000) and increased mortality (p = 0.008).

## Conclusion

Neurosurgical patients with intracranial infection stay longer in ICU, they show more frequent manifestation of other sites infections, and are at greater risk of death. Among the risk factors for intracranial infection a more frequent use of external ventricular drainage is significantly notable.

## Disclosure of interest

None declared

